# p38-MAPK/MSK1-mediated overexpression of histone H3 serine 10 phosphorylation defines distance-dependent prognostic value of negative resection margin in gastric cancer

**DOI:** 10.1186/s13148-016-0255-9

**Published:** 2016-08-31

**Authors:** Shafqat Ali Khan, Ramchandra Amnekar, Bharat Khade, Savio George Barreto, Mukta Ramadwar, Shailesh V. Shrikhande, Sanjay Gupta

**Affiliations:** 1 Epigenetics and Chromatin Biology Group, Gupta Laboratory, Cancer Research Centre, Advanced Centre for Treatment Research and Education in Cancer, Tata Memorial Centre, Kharghar, Navi Mumbai, MH 410210 India; 2 Homi Bhabha National Institute, Training School Complex, Anushakti Nagar, Mumbai, MH 400085 India; 3 Medanta – The Medicity, Gurgaon, Haryana 122001 India; 4 Department of Pathology, Tata Memorial Hospital, Mumbai, MH 400012 India; 5 Department of Surgical Oncology, Gastrointestinal and Hepato-Pancreato-Biliary Service, Tata Memorial Hospital, Mumbai, MH 400012 India

**Keywords:** Gastric cancer, Histone post-translational modification, H3S10 phosphorylation, Resection margin, Prognosis, p38 MAPK/MSK1 pathway

## Abstract

**Background:**

Alterations in histone modifications are now well known to result in epigenetic heterogeneity in tumor tissues; however, their prognostic value and association with resection margins still remain poorly understood and controversial. Further, histopathologically negative resection margins in several cancers have been associated with better prognosis of the disease. However, in gastric cancer, despite a high rate of R0 resection, a considerably high incidence of loco-regional recurrence is observed. We believe alterations of global histone post-translational modifications could help in identifying molecular signatures for defining the true negative surgical resection margins and also the prognosis of gastric cancer patients.

**Results:**

The present study compares the level of H3S10ph among paired tumor and histopathologically confirmed disease-free (R0) proximal and distal surgical resection margin (PRM and DRM) tissue samples of GC patients (*n* = 101). Immunoblotting and immune-histochemical analysis showed a significantly (*p* < 0.01) higher level of H3S10ph in tumor compared to R0 surgical resection margins. Along with tumor, high H3S10ph levels in both PRM and DRM correlated with clinical parameters and poor survival. Interestingly, in the case of PRM and DRM, the association of H3S10ph with poor survival was only found in a patient group with the resection margin distance <4 cm. Further investigations revealed that the increase of H3S10ph in tumor tissues is not due to the change in cell cycle profile but rather an interphase-associated phenomenon. Moreover, an increase in ph-MSK1 and ph-p38 levels in tumor tissues and the decrease in ph-MSK1 and H3S10ph on p38 inhibition in gastric cancer cells confirmed p38-MAPK/MSK1 pathway-mediated regulation of H3S10ph in gastric cancer.

**Conclusions:**

Our study provides the first evidence that p38-MAPK/MSK1-regulated increase of H3S10ph in GC is predictive of a more aggressive cancer phenotype and could help in defining true negative surgical resection margin. Importantly, our data also gave a new rationale for exploration of the use of MSK1 inhibitor in gastric cancer therapy and the combination of histone post-translational modifications, H4K16ac and H4K20me3 along with H3S10ph as epigenetic prognostic markers.

**Electronic supplementary material:**

The online version of this article (doi:10.1186/s13148-016-0255-9) contains supplementary material, which is available to authorized users.

## Background

Gastric cancer (GC) is one of the most common malignancies worldwide. Globally, GC ranks fourth and third in terms of incidence and mortality, respectively [1]. In India, it is one of the most aggressive cancers ranking third and second in terms of incidence and mortality, respectively [2]. Surgery remains the mainstay for cure especially in early cancers, while in locally advanced GC the addition of perioperative/neoadjuvant chemotherapy (NACT) affords a better survival advantage [3]. The current standard practice in GC is to submit the resected stomach for pathological examination to confirm the diagnosis and stage of the tumor as well as to assess the margins of resection using hematoxylin and eosin (H&E) staining. A pathologically negative resection/R0 margin affords the best chance of cure in GC with 5-year survival rates of 13 versus 35 % for positive and negative resection margins, respectively [4]. However, despite an apparently curative surgery, loco-regional recurrence has still been encountered in 87 % of GC patients [5]. Thus, raising the doubt on current pathological techniques used in day to day practice to truly confirm the adequacy of the R0 surgical resection margins. Therefore, there is an urgent need to identify molecular markers and investigate their expression in not only the cancerous tissues but also the surrounding resected (margin) tissue that is apparently free from disease (R0) based on histopathology.

Over the past decade, accumulated evidence indicates towards the association of aberrant covalent histone post-translational modifications (PTMs) with cancer such as loss of acetylation of histone H4 at lysine 16 (H4K16ac) and tri-methylation of histone H4 at lysine 20 (H4K20me3), defined as “histone onco-modifications” [6, 7]. In GC, a high level of tri-methylation of histone H3 at lysine 9 (H3K9me3) was found to be correlated with lympho-vascular invasion, recurrence, poor survival rate, and as an independent prognostic marker [8]. In addition to their role in disease prognosis, epigenetic alterations, specifically DNA methylation, are also reported in field cancerization/defects in various types of cancer, including stomach, liver, colon, lung, breast, kidney, and esophageal [9]. However, the relation of histone PTMs between tumor and resection margin and the regulatory mechanism for their alteration is poorly understood in cancer.

In this study on human GC, we identified phosphorylation of histone H3 at serine 10 (H3S10ph) as a histone PTM with most consistent and significant difference between tumor and negative resection margin. For the first time, our results demonstrate a distance-dependent (≤4 vs >4 cm) relation of H3S10ph of the tumor with both PRM and DRM and also it correlates with the prognosis of GC patients. Further, we report that phosphorylation of H3S10 in GC is mediated by mitogen- and stress-activated protein kinase-1 (MSK1) through the p38-MAPK pathway.

## Methods

### Tissue samples and histopathological analysis

Frozen tissue sections (*n =* 36) and formalin-fixed paraffin-embedded (FFPE) tissue blocks (*n =* 115) of the tumor (T), PRM, and DRM from each gastric adenocarcinoma patients were obtained. All the patients were operated between 2009 and 2012 at Tata Memorial Hospital, Mumbai, India. Histopathological analysis including determination of tumor content (% of tumor cells) was done by a blinded gastrointestinal pathologist. Based on the histopathological analysis, frozen tumor tissues with ≥70 % tumor content, FFPE tumor tissues with ≥10 % tumor content, and negative resection margins (without any tumor cell) were included in the study. Finally, the study was conducted on paired tumor, PRM, and DRM frozen tissues (*n =* 10), and FFPE tissue blocks (*n =* 101). The protocol of the study was reviewed and approved by the Institutional Review Board and Ethics Committee (project number-466). All patients provided a written informed consent.

### Immunohistochemistry

Immunohistochemical (IHC) staining was performed using VECTASTAIN® ABC kit (Vector Lab-P6200). FFPE tissue blocks were sectioned at a thickness of 4 μm and mounted on poly-l-lysine-coated glass slides. The sections were deparaffinized through a graded series of xylene and rehydrated through a graded series of absolute ethanol to distilled water. Endogenous peroxidase was quenched with 3 % hydrogen peroxide in methanol at room temperature for 30 min in the dark. Microwave antigen retrieval was carried out with 0.01 M Sodium citrate buffer (pH 6). Anti H3S10ph (Abcam-1776), H3K16ac (Millipore-07-329), H4K20me3 (Abcam-9053), and ph-MSK1 (Abcam-32190) antibodies were applied for 16 h at 4 °C at the dilution of 1:100. Immunoreactive proteins were chromogenically detected with diaminobenzidine (DAB; Sigma-D5537). The sections were counterstained with Harris’s hematoxylin, dehydrated, and mounted. In parallel, control staining was performed without adding a primary antibody.

### Evaluation of immunohistochemistry

The nuclear IHC staining for all the antibodies were scored using H-score which is based on intensity of staining (ranges from zero to three) and percentage of stained cells using the following formula: H-score = [(0 × % of cells with staining intensity of zero) + (1 × % of cells with staining intensity of one) + (2 × % of cells with staining intensity one) + (3 × % of cells with staining intensity two)]. H-score was further divided into three groups: (i) 0–100: low level, (ii) 100–200: intermediate level, and (iii) 200–300: high level. The IHC staining was examined by two independent researchers (SAK and MR), one of whom is a senior consultant pathologist (MR). Both the researchers were blinded to all clinicopathological and outcome variables.

### Cell culture, transfection, and treatment

GC cell lines, AGS (CRL-1739) and KATOIII (HTB-103), were procured from ATCC. AGS and KATOIII were cultured in RPMI1640 (Invitrogen) and F12K (Himedia) media, respectively, at 37 °C with 5 % CO_2_ supplemented with 10 % FBS, 100 U/ml penicillin, and 100 mg/ml streptomycin (Sigma). AGS cells were transfected using CaCl_2_ method with 10 μg of the pCMV-flag-MSK1 construct. AGS and KATOIII cells were treated for 1 h with chemical inhibitors against ph-p38 (SB203580, Calbiochem), ph-ERK (PD98059, Calbiochem), and ph-MSK1 (H89, Millipore) at 10 and 20 μM concentrations for 1 h, respectively.

### Total RNA isolation and RT-PCR

Total RNA was extracted (Thermo scientific-0731) from 25 mg of the frozen tumor and resection margin (PRM or DRM) tissues with the maximum distance from the site of the tumor. Total RNA (1 μg) was used for cDNA synthesis (Fermentas-K1632) using random hexamers. RT-PCR amplification was done using specific primers for *c-Jun* (F:CCCCAAGATCCTGAAACAGA, R:TCCTGCTCATCTGTCACGTT), *c-Fos* (F:CGGGGATAGCCTCTCTTAC, R:CCCTTCGGATTCTCCTTTTC), cyclin-E1 (F:AGCGGTAAGAAGCAGAGCAG, R:TTTGATGCCATCCACA GAAA), cyclin-B1 (F:CGGGAAGTCACTGGAAACAT, R:CCGACCCAGACCAAAGTTTA), cyclin-D1 (F:GATCAAGTGTGACCCGGACT, R:AGAGATGGAAGGGGGAAAGA), and 18S rRNA (F:AAACGGCTACCACATCCAAG, R:CCTCCAATGGATCCTCGTTA) with an initial denaturation step at 95 °C for 2 min, followed by 15 cycles of denaturation at 95 °C for 45 s, primer annealing at 55 °C for 30 s, primer extension at 72 °C for 30 s, and a final extension at 72 °C for 10 min. Amplified products were resolved on 1.5 % agarose gels and visualized by EtBr staining.

### Preparation of total cell lysate, nucleo-cytosolic fraction, and histones

Cells were harvested from 90-mm plates and lysed in 200 μl of Laemmli buffer to prepare the total cell lysate (TCL). Nucleo-cytosolic fraction (NCF) was prepared by homogenizing 100 mg of frozen tissue in 2 ml of ice-cold lysis buffer (20 mM Tris-Cl pH 8, 2 mM EDTA pH 8, 10 mM EGTA, 5 mM MgCl_2_, 0.1 % TritonX-100, 1 mM sodium orthovanadate, 1 mM sodium fluoride, 20 mM β-glycerophosphate, 1 mM DTT, 1 mM PMSF, 10 μg/ml leupeptin, 10 μg/ml aprotinin), and for cell lines, 2 ml of ice-cold MKK lysis buffer (10 mM Tris-Cl, 1 mM EDTA, 1 mM EGTA, 1 % Triton X-100, 10 μg/ml leupeptin, 10 μg/ml aprotinin, 1 mM PMSF, 1 mM sodium orthovanadate, 10 mM sodium fluoride, 10 mM β-glycerophosphate) [10] was used for cells harvested from a 90-mm plate. Both the homogenates were then kept at 4 °C for 30 min with intermittent mixing and then was clarified by centrifugation at 16,000 rpm for 30 min. The supernatant was collected as NCF and stored at −20 °C until it is required, and the pellet was used for histone isolation by acid extraction method [11]. For the preparation of NCF and histones, along with tumor, resection margin (PRM or DRM) tissues with the maximum distance from the site of the tumor were used.

### Electrophoresis and immunoblotting

TCL and NCF, and histones were resolved on 10 and 18 % polyacrylamide SDS-PAGE, respectively, and transferred to PVDF membrane. Proteins on PVDF membrane were hybridized with anti-H3 (Upstate-06-755; 1:2000 dilution), H4 (Millipore-07-108; 1:4000 dilution), H3S10ph (Millipore-06-570; 1:7000 dilution), H4K16ac (Millipore-07-329; 1:8000 dilution), H4K20me3 (Abcam-9053; 1:4000 dilution), β-actin (Sigma-A5316; 1:10,000 dilution), MSK1 (Santacruz-9392; 1:2000 dilution), ph-MSK1 (Abcam-31190; 1:3000 dilution), ERK1/2 (Santacruz-292838; 1:2000 dilution), ph-ERK (Cell signaling-9910; 1:2000 dilution), p38 (Santacruz-728; 1:2000 dilution), ph-p38 (Cell signaling-9910; 1:2000 dilution), and anti-flag (Sigma-F3165; 1:5000 dilution). Signal was visualized using horseradish peroxidase-conjugated anti-rabbit/mouse secondary antibody and ECL plus chemiluminescence kit (Amersham). Wherever required, the densitometry analysis was done on immunoblot and membrane to determine their mean intensities using ImageJ software. For native proteins, mean intensity of immunoblot was normalized with the stained PVDF membrane; for phosphorylated forms, mean intensity of immunoblot was normalized with immunoblot of native proteins. The resulted value was used to express their mean relative levels in resection margin and tumor.

### Immunofluorescence microscopy

Cells grown on glass coverslips were fixed with 4 % paraformaldehyde for 20 min. Cells were then permeabilized in PBS containing 0.5 % Triton X-100 for 20 min at RT and then blocked with PBS containing 3 % BSA and 0.1 % NP-40 for 1 h. Next, cells were incubated with a primary antibody against H3S10ph (Millipore-06-570) and ph-MSK1 (Abcam-31190) and appropriate secondary antibodies for 2 h each. Dilution of primary (1:100) and secondary antibodies (Alexa-568 or Alexa-488; Cell signaling; 1:300 dilution) was made in blocking buffer. After the addition of secondary antibody, all the steps were performed in the dark and at room temperature. Finally, coverslips were mounted in VECTASHIELD (Vector lab). Fluorescence intensity was analyzed using a fluorescence microscope (IX81; Olympus, Japan).

### Cell cycle analysis

Frozen tissue (50 mg) was powdered using mortar and pestle in liquid nitrogen. The powder was homogenized in 2 ml of nuclear buffer A (15 mM Tris-Cl pH 7.5, 60 mM KCl, 15 mM NaCl, 2 mM EDTA, 0.5 mM EGTA, 0.34 M sucrose, 0.15 mM β-ME, 0.15 mM spermine, and 0.5 mM spermidine) using a Dounce homogenizer. In the case of cell lines, cells were trypsinized and harvested in PBS. Tissue homogenate and cells were then centrifuged (5000 rpm for 15 min at 4 °C) to pellet nuclei and cells, respectively; the supernatant was discarded. The pellet was then fixed with 70 % chilled ethanol and stored at −20 °C. When required, tissue nuclei/cells were washed with PBS and suspended in 500 μl of PBS containing 0.1 % TritonX-100 and 100 μg/ml of RNaseA and incubated at 37 °C for 30 min. After incubation, propidium iodide (25 μg/ml) was added and nuclei were incubated at 37 °C for 30 min. DNA content analysis was carried out in a FACSCalibur flow cytometer (BD Biosciences). Cell cycle analysis was performed using the Mod-fit software.

### Mitotic index analysis

Based on the morphology of nuclei, mitotic cells were counted in 10 consecutive high power fields (HPF; ×40) of H&E-stained tissue (tumor and resection margin) section slides. Average of mitotic cells from 10 HPF was represented as mitotic index. H&E staining was done as per the standard protocol on 4-μm-thick FFPE tissue sections.

### Statistical analysis

To test the statistical significance of paired and unpaired resection margin and tumor tissues, Wilcoxon matched pair and Mann-Whitney test were used, respectively. To test whether variables differed across groups, we used the chi-square test. Chi-square analysis was used to find the correlation between H3S10ph levels of the tumor, PRM, and DRM tissues and clinical parameters. Survival curves were plotted using the Kaplan-Meier method, and the significance of the differences between the survival curves was determined using a univariate log-rank test. To test the statistical independence and significance of predictors, a multivariate survival analysis was performed using the Cox proportional hazard regression model. All *p* values were two-sided, and *p <* 0.05 was considered significant. All statistical analyses were performed with GraphPad and/or SPSS software.

## Results

### Level of H3S10ph in tumor and resection margin tissues of GC

**Fig. 1:**
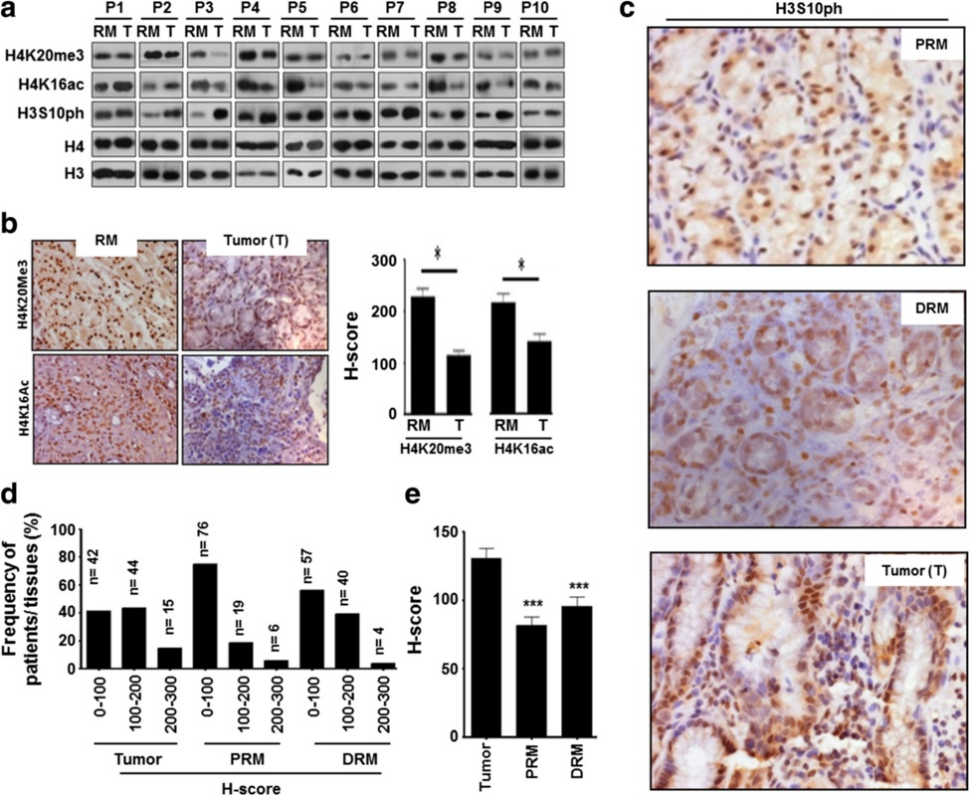
H3S10ph level in paired tumors and negative PRM and DRM GC tissue samples: **a** immunoblot analysis of H3S10ph, H4K16ac, and H4K20me3 in freshly resected paired tumor and resection margin tissues (*n =* 10). **b** Representative image (*left panel*, ×40) and comparison of mean H-score (*right panel*) for H4K16ac and H4K20me3 immunostaining (*n =* 10). **c** Representative images (×40) of H3S10ph immunostaining (*n =* 101). **d** Comparison of frequency distribution with low (H-score 0–100), intermediate (H-score 100–200), and high (H-score 200–300) levels of H3S10ph immunostaining (*n =* 101). **e** Comparative mean H-score of H3S10ph immunostaining (*n =* 101). *GC* gastric cancer, *PRM* proximal resection margin, *DRM* distal resection margin, *RM* resection margin either PRM or DRM with maximum distance from the site of the tumor, *P* patient. Statistical tests are done by using Wilcoxon matched pairs test. *p < 0.05, **p < 0.01, ***p<0.001

Histones were prepared from freshly resected paired (*n =* 10) tumor and R0 resection margin (RM) tissues of GC patients, for a pilot study. Histones and their respective paraffin blocks were subjected to immunoblotting and IHC analysis with site-specific acetylation, methylation, and phosphorylation marks of histone H3 and H4 (data not shown). H3S10ph showed a most consistent (9/10 patients) increase in tumor compared to resection margin tissues in immunoblot analysis (Fig. 1a). Further, the loss of H4K16ac and H4K20me3 is a hallmark of the tumor [7]; however, it was not reported in paired GC tissue samples. Our immunoblot and IHC analysis in the paired tissues confirmed the decrease of H4K16ac (8/10 patients) and H4K20me3 (8/10 patients) in GC as well (Fig. 1a, b and ​Additional file 1: Figure S1).

The status of H3S10ph was further studied in a validation set (*n =* 101) among tumor and histopathologically negative PRM and DRM tissues using IHC. IHC analysis showed a high level of H3S10ph in tumor compared to both the resection margins (Fig. 1c). H-score-based analysis of frequency distribution of tumor and PRM and DRM tissue samples of GC patients showed 76, 57, and 42; 19, 40, and 44; and 6, 4, and 15 samples with a low, intermediate and high level of H3S10ph, respectively (Fig. 1d). Further, comparison of H-score showed a significant high level of H3S10ph in tumor compared to PRM (*p <* 0.001) and DRM (*p <* 0.001) tissues (Fig. 1e).

### Correlation of H3S10ph levels of tumor, PRM, and DRM with clinicopathological variable of GC patients

Chi-square analysis was used to find a correlation between H3S10ph levels of the tumor, PRM, and DRM tissues, and clinical parameters (Table 1). H3S10ph levels of tumor tissues showed a significant positive correlation with the World Health Organization (WHO) classification (*p* = 0.0001), T stage (*p* = 0.005), pTNM stage (*p* = 0.016), and recurrence (*p* = 0.034). Interestingly, except recurrence, H3S10ph levels of PRM and DRM tissues also showed a significant positive correlation with the same clinical parameters as tumor tissues; WHO classification (*p =* 0.008 and 0.0001 for PRM and DRM, respectively), T stage (*p =* 0.001 and 0.003 for PRM and DRM, respectively), and pTNM stage (*p =* 0.015 and 0.037 for PRM and DRM, respectively). Only DRM showed a significant correlation with recurrence (*p =* 0.012).

**Table 1:** Correlation between H3S10 phosphorylation levels of tumor, PRM, and DRM with clinicopathological variables

Total (*n =* 101)	H3S10 phosphorylation level of tumor			*p* value	H3S10 phosphorylation level of PRM			*p* value	H3S10 phosphorylation level of DRM			*p* value
	Low (%), *n =* 42	Inter. (%), *n =* 44	High (%), *n =* 15		Low (%), *n =* 76	Inter. (%), *n* = 19	High (%), *n =* 6		Low (%), *n =* 57	Inter. (%), *n =* 40	High (%), *n =* 4	
Age (years)												
≤50	15 (35.7)	18 (40.9)	6 (40.0)	0.879^‡^	31 (40.8)	6 (31.6)	2 (33.3)	0.734^‡^	21 (36.8)	16 (40.0)	2 (50.0)	0.849^‡^
>50	27 (64.3)	26 (59.1)	9 (60.0)		45 (59.2)	13 (68.4)	4 (66.7)		36 (63.2)	24 (60.0)	2 (50.0)	
Sex												
Male	29 (69.0)	32 (72.7)	9 (60.0)	0.652^‡^	53 (69.7)	11 (57.9)	6 (100.0)	0.148^‡^	40 (70.2)	26 (65.0)	4 (100.0)	0.343^‡^
Female	13 (31.0)	12 (27.7)	6 (40.0)		23 (30.3)	8 (42.1)	0 (0.0)		17 (29.8)	14 (35.0)	0 (0.0)	
WHO classification												
WD	2 (4.8)	0 (0.0)	0 (0.0)	*0.0001* ^‡^	2 (2.6)	0 (0.0)	0 (0.0)	*0.008* ^‡^	2 (3.5)	0 (0.0)	0 (0.0)	*0.0001* ^‡^
MD	22 (52.4)	3 (6.8)	0 (0.0)		24 (31.6)	1 (5.3)	0 (0.0)		23 (40.4)	2 (5.0)	0 (0.0	
PD	16 (38.1)	40 (40.9)	7 (46.7)		44 (57.9)	16 (84.2)	3 (50.0)		29 (50.9)	33 (82.5)	1 (25.0)	
SRC	2 (4.8)	1 (2.3)	8 (53.3)		6 (7.9)	2 (10.5)	3 (50.0)		3 (5.3)	5 (12.5)	3 (75.0)	
T stage												
T1	9 (21.4)	4 (9.1)	1 (6.7)	*0.005* ^†^	13 (17.1)	1 (5.3)	0 (0.0)	*0.001* ^†^	11 (19.3)	3 (7.5)	0 (0.0)	*0.003* ^†^
T2	11 (26.2)	10 (22.7)	3 (20.0)		22 (28.9)	2 (10.5)	0 (0.0)		14 (24.6)	10 (25.0)	0 (0.0	
T3	16 (38.1)	20 (45.5)	2 (13.3)		26 (34.2)	10 (52.6)	2 (33.3)		23 (40.4)	15 (37.5)	0 (0.0)	
T4	6 (14.3)	10 (22.7)	9 (60.0)		15 (19.7)	6 (31.6)	4 (66.7)		9 (15.8)	12 (30.0)	4 (0.0)	
Lymph node metastasis												
Absent	20 (47.6)	27 (61.4)	10 (66.7)	0.136^†^	42 (55.3)	10 (52.6)	5 (83.3)	0.385^†^	30 (52.6)	24 (60.0)	3 (75.0)	0.311^†^
Present	22 (52.4)	17 (38.6)	5 (33.3)		34 (44.7)	9 (47.4)	1 (16.7)		27 (47.4)	16 (40.0)	1 (25.0)	
pTNM stage												
I	14 (33.3)	7 (15.9)	1 (6.7)	*0.016* ^†^	20 (26.3)	2 (10.5)	0 (0.0)	*0.015* ^†^	17 (29.8)	5 (12.5)	0 (0.0)	*0.037* ^†^
II	15 (35.7)	22 (50.0)	6 (40.0)		33 (43.4)	8 (42.1)	2 (33.3)		22 (38.6)	20 (50.0)	1 (25.0)	
III	13 (31.0)	14 (31.8)	7 (46.7)		22 (28.9)	8 (42.1)	4 (66.7)		17 (29.8)	14 (35.0)	3 (75.0)	
IV	0 (0.0)	1 (2.3)	1 (6.7)		1 (1.3)	1 (5.3)	0 (0.0)		1 (1.8)	1 (2.5)	0 (0.0)	
Recurrence												
Absent	32 (76.2)	28 (63.6)	7 (46.7)	*0.034* ^†^	54 (71.1)	8 (42.1)	5 (83.3)	0.351^†^	43 (75.4)	23 (57.5)	1 (25.0)	*0.012* ^†^
Present	10 (23.8)	16 (36.4)	8 (53.3)		22 (28.9)	11 (57.9)	1 (16.7)		14 (24.6)	17 (42.5)	3 (75.0)	
Treatment modality												
Surgery	24 (57.1)	21 (47.7)	12 (80.0)	0.093^‡^	43 (56.6)	11 (57.9)	3 (50.0)	0.943^‡^	28 (49.1)	26 (65.0)	3 (75.0)	0.087^‡^
NACT + Surgery	18 (42.9)	23 (52.3)	3 (20.0)		33 (43.4)	8 (42.1)	3 (50.0)		29 (50.9)	14 (35.0)	1 (25.0)	

All three columns are compared in each category: ^†^chi-square test by two-sided linear-by-linear association; ^‡^chi-square test by two-sided Fisher’s exact test. Italics indicates values that are statistically significant (<0.05)

*Int.* intermediate, *PRM* proximal resection margin, *DRM* distal resection margin

### Correlation of H3S10ph levels of tumor and resection margins with survival of GC patients

**Fig. 2:**
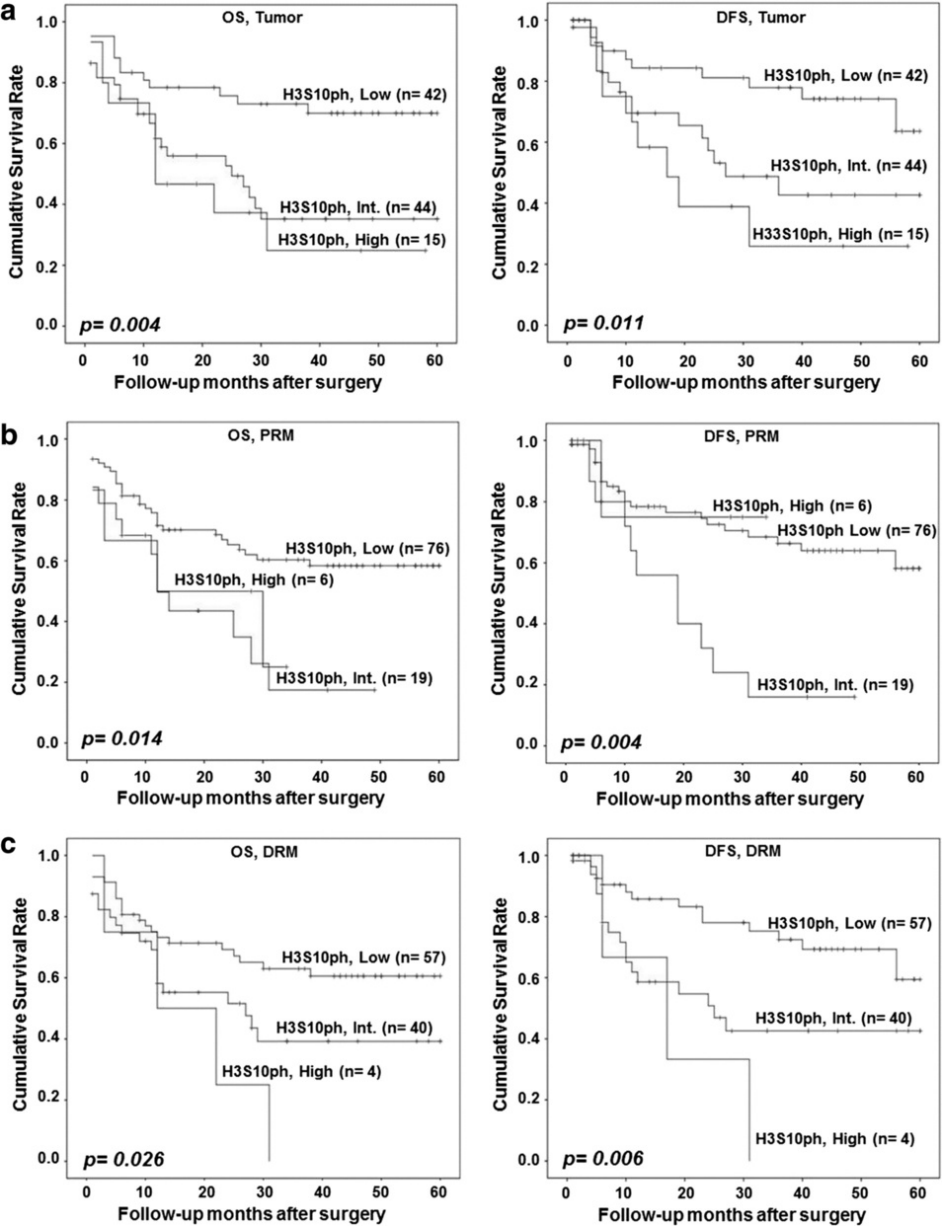
Effect of H3S10ph levels of tumor, PRM, and DRM on GC patients’ survival. Kaplan-Meier survival analysis was done according to H3S10ph staining H-score: low (0–100), intermediate (100–200), and high (200–300). High level of H3S10P of tumor and PRM and DRM is associated with both poor overall survival (OS) and disease-free survival (DFS). **a** OS and DFS based on H3S10P levels of tumor tissues, **b** OS and DFS based on H3S10P levels of PRM tissues, **c** OS and DFS based on H3S10P levels of DRM tissues. *GC* gastric cancer, *PRM* proximal resection margin, *DRM* distal resection margin, *Int.* intermediate. Comparison was done by log-rank test. *p <* 0.05 was considered as significant

Overall survival (OS) and disease-free survival (DFS) rate among groups with the low, intermediate, and high level of H3S10ph was compared by log-rank test/Kaplan-Meier survival analysis (Fig. 2). Analysis showed a significant negative correlation of H3S10ph levels of tumor (*p =* 0.004 and 0.011), PRM (*p =* 0.014 and 0.004), and DRM (*p =* 0.026 and 0.006) with OS and DFS, respectively (Fig. 1a–c). Moreover, H3S10ph levels of the tumor, but not the PRM and DRM, were found to be independent predictors of overall survival (Additional file 2: Table S1). Therefore, together, data of this and previous sections confirm the association of a high level of H3S10ph of resection margins along with tumor tissues with poor prognosis of GC.

### Relation of H3S10ph levels of resection margins and their distance from the site of the tumor in GC

**Fig. 3:**
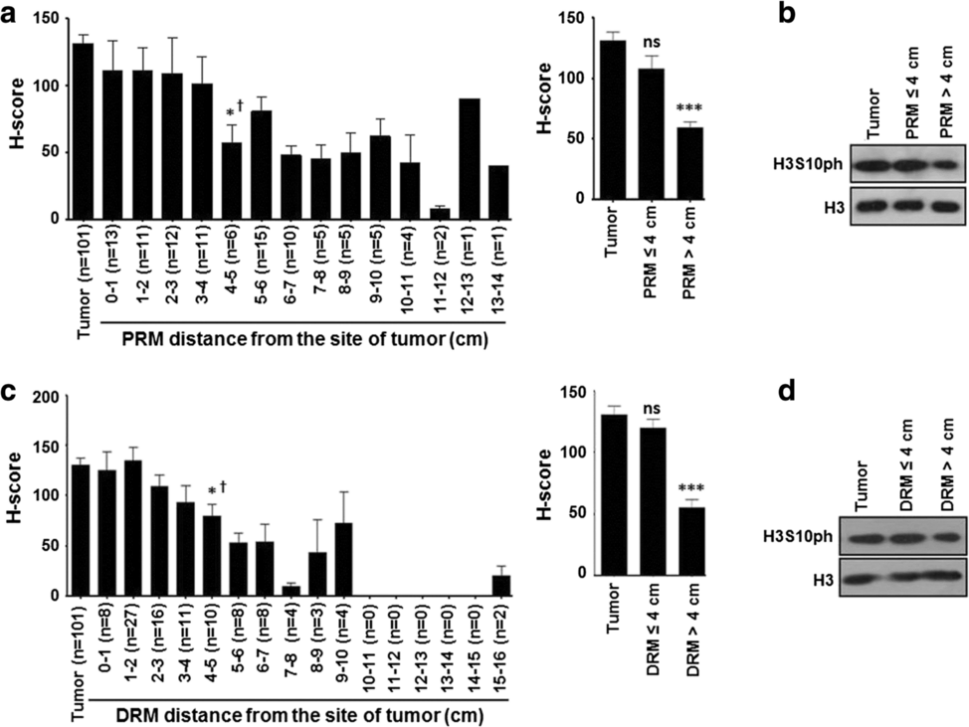
Association of H3S10ph with the distance of negative resection margins in GC. **a**, **c** Resection margins were grouped as per their distance from the site of the tumor with 1 cm interval and mean H-score of H3S10ph immunostaining of each group was compared with tumor (*left panel*). In case of both PRM and DRM, analysis showed a significant decrease in H3S10ph levels as the margin length reaches more than 4 cm (*left panel*). Comparison of mean H-score of H3S10ph immunostaining of all resection margins with a margin distance ≤4 and >4 cm with tumor confirms the significant reduction of H3S10ph if the margin distance is >4 cm (*right panel*). **b**, **d** Confirmation of reduction of H3S10ph, if the margin length is >4 cm by immunoblotting. *GC* gastric cancer, *PRM* proximal resection margin, *DRM* distal resection margin. Statistical tests are done by using Mann-Whitney test (^†^) and Wilcoxon matched pairs test. *p < 0.05, **p < 0.01, ***p<0.001

Our observation of the low level of H3S10ph in resection margin compared to tumor tissues led us to examine whether the decrease had any relation with the distance of resection margin from the site of the tumor. To answer this, we first grouped the resection margin samples as per their distance from the tumor site and compared the mean H-score of H3S10ph immunostaining of each group with the mean H-score of tumor samples (Fig. 3). For both PRM and DRM, a significant reduction in HS10ph (*p <* 0.05) was observed for patient’s group with resection margin distance >4 cm (Fig. 3a, b, left panel). Further, patients were divided into two groups based on the distance of resection margin ≤4 or >4 cm and their mean H-scores were compared with the tumor tissues. Interestingly, further analysis showed H3S10ph levels of resection margins with the distance ≤4 cm were almost equal to those of the tumor tissues; however, resection margins with >4 cm showed a significant (*p <* 0.001) reduction in both PRM and DRM (Fig. 3a, b, middle panel). Additionally, immunoblot analysis also confirmed the reduction of H3S10ph levels of resection margins if the distance is >4 cm from the site of the tumor (Fig. 3a, b, right panel).

### Effect of resection margin distance on prognostic value of H3S10ph in GC

To investigate the effect of resection margin distance from the site of the tumor on the prognostic value of H3S10ph, its association with clinicopathological variables and survival was compared between the group with the resection margin ≤4 and >4 cm. Chi-square analysis (Table 2) showed a positive correlation of H3S10ph levels with WHO classification (*p =* 0.001), T stage (*p =* 0.002), and TNM stage (*p =* 0.023) for the patients with resection margin ≤4 cm. In case of DRM, chi-square analysis showed a positive correlation of H3S10ph levels with WHO classification (*p =* 0.0001) and T stage (*p =* 0.009) and recurrence (*p =* 0.031) for the patients with resection margins ≤4 cm. For both the resection margins, no correlation was found for patients with >4 cm resection margin distance.

**Table 2:** Correlation between H3S10ph levels of PRM and DRM, ≤4 vs >4 cm

Total (*n =* 101)	H3S10 phosphorylation level of PRM ≤4 cm (*n =* 48)			*p* value	H3S10 phosphorylation level of PRM >4 cm (*n =* 53)			*p* value
	Low (%), *n =* 28	Int. (%), *n =* 14	High (%), *n =* 6		Low (%), *n =* 48	Int. (%), *n =* 5	High (%), *n =* 0	
WHO classification								
WD	1 (3.6)	0 (0.0)	0 (0.0)	*0.001* ^‡^	1 (2.1)	0 (0.0)	0 (0.0)	0.632^‡^
MD	14 (50.0)	1 (7.1)	0 (0.0)		10 (20.8)	0 (0.0)	0 (0.0)	
PD	12 (42.9)	12 (85.7)	3 (50.0)		32 (66.7)	4 (80.0)	0 (0.0)	
SRC	1 (3.6)	1 (7.1)	3 (50.0)		5 (10.4)	1 (20.0)	0 (0.0)	
T stage								
T1	6 (21.4)	1 (7.1)	0 (0.0)	*0.002* ^†^	7 (14.6)	0 (0.0)	0 (0.0)	0.121^†^
T2	10 (35.7)	2 (14.3)	0 (0.0)		12 (25.0)	0 (0.0)	0 (0.0)	
T3	8 (28.6)	7 (50.0)	2 (33.3)		18 (37.5)	3 (60.0)	0 (0.0)	
T4	4 (14.3)	4 (28.6)	4 (66.7)		11 (22.9)	2 (40.0)	0 (0.0)	
pTNM stage								
I	10 (35.7)	2 (14.)	0 (0.0)	*0.023* ^†^	10 (20.8)	0 (0.0)	0 (0.0)	0.068^†^
II	10 (35.7)	6 (42.9)	2 (33.3)		23 (47.9)	2 (40.0)	0 (0.0)	
III	8 (28.6)	6 (42.9)	4 (66.7)		14 (29.2)	2 (40.0)	0 (0.0)	
IV	0 (0.0)	0 (0.0	0 (0.0)		1 (2.1)	1 (10.0)	0 (0.0)	
Recurrence								
Absent	20 (71.4)	8 (57.1)	5 (57.1)	0.956^†^	34 (70.8)	2 (40.0)	0 (0.0)	0.193^†^
Present	8 (28.6)	6 (42.9)	1 (16.7)		14 (29.2)	3 (50.0)	0 (0.0)	
Total (*n =* 101)	H3S10 phosphorylation level of DRM ≤4 cm (*n =* 62)			*p* value	H3S10 phosphorylation level of DRM >4 cm (*n =* 39)			*p* value
	Low (%), *n =* 24	Int. (%), *n =* 34	High (%), *n =* 4		Low (%), *n =* 33	Int. (%), *n =* 6	High (%), *n =* 0	
WHO classification								
WD	1 (4.2)	0 (0.0)	0 (0.0)	*0.0001* ^‡^	1 (3.0)	0 (0.0)	0 (0.0)	0.6^‡^
MD	10 (41.7)	1 (2.9)	0 (0.0)		13 (39.4)	1 (16.7)	0 (0.0)	
PD	12 (4.2)	29 (85.3)	1 (25.0)		17 (51.5)	4 (66.7)	0 (0.0)	
SRC	1 (4.2)	4 (11.8)	3 (75.0)		2 (6.1)	1 (16.7)	0 (0.0)	
T stage								
T1	5 (20.8)	3 (8.8)	0 (0.0)	*0.009*	6 (18.2)	0 (0.0)	0 (0.0)	0.287^†^
T2	6 (25.0)	8 (23.5)	1 (25.0)		8 (24.2)	2 (33.3)	0 (0.0)	
T3	9 (37.5)	13 (38.2)	3 (75.0)		14 (42.4)	2 (33.3)	0 (0.0)	
T4	4 (16.7)	10 (29.4)	0 (0.0)		5 (15.2)	2 (33.3)	0 (0.0)	
pTNM stage								
I	5 (20.8)	5 (14.7)	0 (0.0	0.361^†^	12 (36.4)	0 (0.0)	0 (0.0)	0.107^†^
II	9 (37.5)	17 (50.0)	1 (25.0)		13 (39.4)	3 (50.0)	0 (0.0)	
III	10 (41.7)	11 (32.4)	3 (75.0)		7 (21.2)	3 (50.0)	0 (0.0)	
IV	0 (0.0)	1 (2.9)	0 (0.0)		1 (3.0)	0 (0.0)	0 (0.0)	
Recurrence								
Absent	19 (79.2)	13 (38.2)	1 (25.0)	*0.031* ^†^	24 (72.7)	4 (66.7)	0 (0.0)	0.063^†^
Present	5 (20.8)	21 (61.8)	3 (75.0)		9 (27.3)	2 (33.3)	0 (0.0)	

All three columns are compared in each category: ^†^chi-square test by two-sided linear-by-linear association; ^‡^chi-square test by two-sided Fisher’s exact test. Italics indicates values that are statistically significant (<0.05)

In the case of OS, patients with PRM ≤4 cm showed a significant (*p* = 0.003) difference among the group of high, intermediate, and low levels of H3S10ph (Additional file 3: Figure S2A) and no difference was observed in the case of DRM (Additional file 2: Figure S1C). However, in the case of DFS, distance seems to have no effect as patients with both ≤4 or >4 cm resection showed significant difference in survival among the group of high, intermediate, and low levels of H3S10ph for both PRM (*p =* 0.028 vs 0.006) and DRM (*p =* 0.041 vs 0.005). Moreover, when the patients were grouped just based on the distance of the resection margin from the site of the tumor (≤4 or >4 cm), no significant difference was observed in the case of both OS and DFS (Additional file 4: Figure S3).

Taken together, these data indicate that the distance of resection margin is an important factor in GC prognosis and H3S10ph could be a potential biomarker in predicting the association between distance of resection margin and clinical parameters. However, H3S10ph cannot be used to predict the survival difference based on the distance of the resection margin for both PRM and DRM.

### Association of an increase of H3S10ph with the phase of cell cycle in GC

**Fig. 4:**
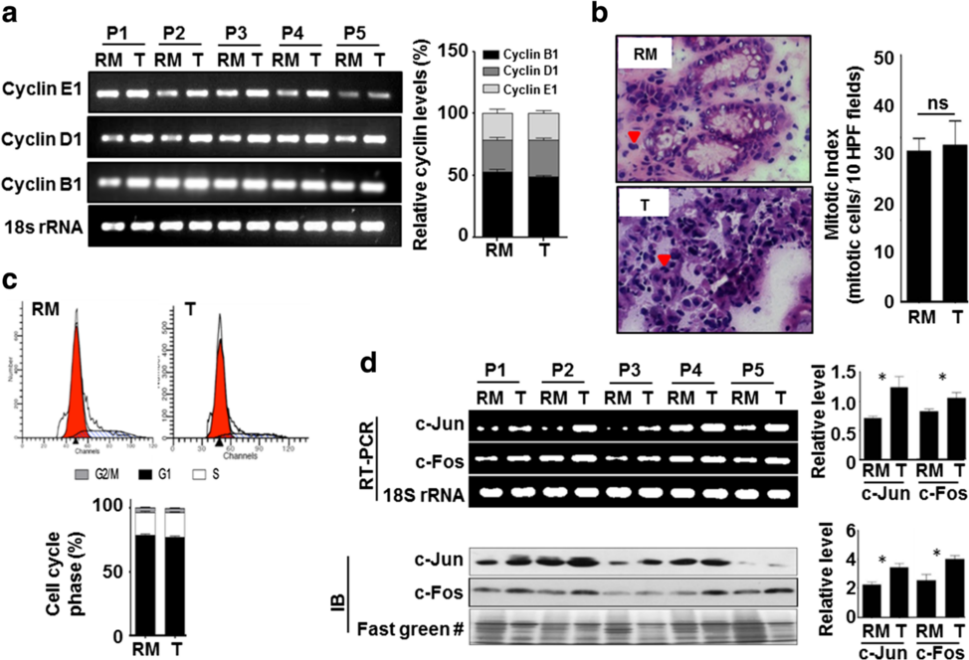
Association of cell cycle profile with GC tumor and resection margin tissues. **a** RT-PCR analysis shows a high mRNA level of cyclin B1, D1, and E1 in tumor than resection margin tissues (*left panel*). mRNA level of cyclins were normalized with 18S rRNA, combined % was calculated for each cyclin in tumor and resection margin tissues, and their relative % levels were compared showing no difference in cell cycle profile of tumor and resection margin tissues (*right panel*). **b** *Arrow heads* showing mitotic cells in H&E-stained resection margin and tumor tissue section image (×40, *left panel*). On H&E-stained tissue sections, mitotic index was calculated for paired samples (*n =* 40), comparison between tumor and resection margin tissues showing no significant difference (*right panel*). **c** Flow cytometry-based cell cycle profile showed most of the cells of both tumor and resection margin tissues are in G1 phase (*upper panel*). Comparison of mean % (*n =* 10) of G2/M, G1, and S phases of cell cycle showed no difference in cell cycle profile of tumor and resection margin tissues (*lower panel*). **d** RT-PCR (*left upper panel*) and immunoblot (*left lower panel*) analyses showed a high level of immediate early genes, *c-jun* and *c-fos*, in tumor than resection margin tissues. After normalization, comparison of relative level also showed significant increase of *c-jun* and *c-fos* in tumor tissues, both at transcript (*right upper panel*) and protein (*right lower panel*) level. *GC* gastric cancer, *RM* resection margin either PRM or DRM with maximum distance from the site of the tumor, *T* tumor, *P* patient. #Fast green-stained PVDF membrane used in Fig. 5a as well. Statistical tests are done by using Wilcoxon matched pairs test. *p < 0.05, **p < 0.01, ***p<0.001

H3S10ph is a very dynamic histone mark and its level changes throughout the cell cycle with the highest level in mitotic phase and the lowest level in the interphase of the cell cycle [12, 13]. Therefore, to determine whether the increase of H3S10ph in GC is dependent on the cell cycle profile of the tissues samples, cyclin levels, mitotic index, and cell cycle profile of the tumor and resection margin tissues were studied (Fig. 4). Cyclin B1, D1, and E1 levels are known to peak at the time of G2/M phase transition, mid-S phase, and G1/S phase transition, respectively. RT-PCR analysis showed the increase in the messenger RNA (mRNA) levels of all the cyclins in tumor than the resection margin tissues; however, no changes were observed in their ratios between the tumor and resection margin tissues (Fig. 4a). The mitotic index also did not show any significant increase in mitotic cells in tumor compared to resection margin tissues (Fig. 4b). Flow cytometry-based cell cycle analysis of tissue samples showed an equal percentage of G1, S, and G2/M cells in the tumor and resection margin tissues with a maximum population of cells in the G1 phase (Fig. 4c). These results indicate that the observed increase of H3S10ph in GC is not because of the enrichment of cells in any cell cycle phase in tumor compared to resection margin tissues.

In the mitotic phase, H3S10ph is associated with chromatin condensation and transcription silencing while in the interphase of cell cycle an increase of H3S10ph is associated with chromatin relaxation and transcription upregulation of mainly immediate early (IE) genes [12, 13]. Cell cycle analysis revealed about 80 % cells of the tumor and resection margin tissues were in G1 phase (Fig. 4c). Therefore, to determine whether the increase in H3S10ph in GC is an interphase-associated phenomenon or not, we checked the levels of IE genes (*c-jun* and *c-fos*) using RT-PCR and immunoblotting. The data showed an increase in the levels of *c-jun* and *c-fos* in tumor compared to resection margin tissues (Fig. 4d). Therefore, taken together, these data confirm that increase in H3S10ph levels in GC is not due to the alteration in the cell cycle phase, but an interphase-associated phenomenon.

### MSK1 phosphorylates H3S10 through p38-MAPK pathway in GC

**Fig. 5:**
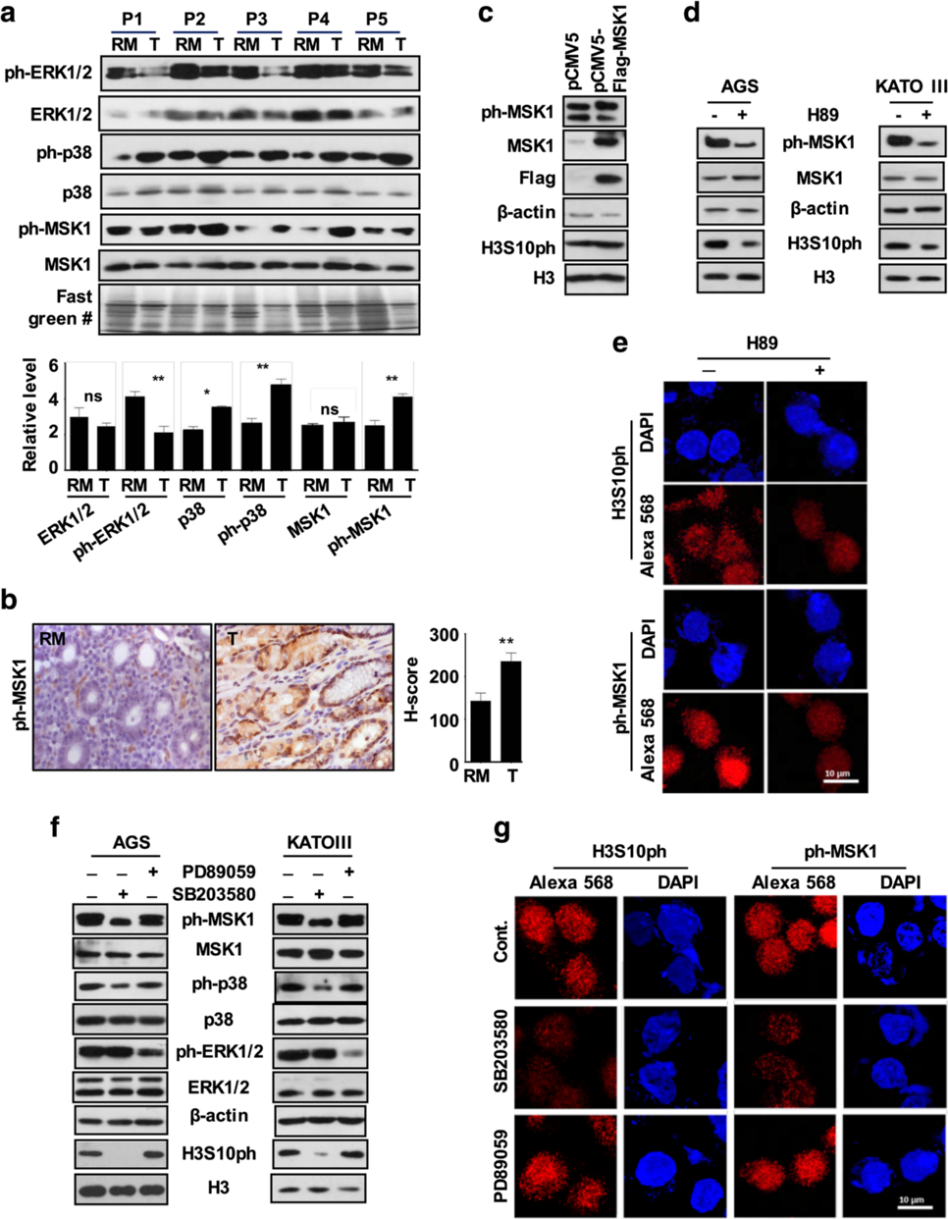
Regulatory mechanism for differential levels of H3S10ph in GC. **a** Immunoblot analysis (*upper panel*) of paired tissue (*n =* 5) and densitometry-based analysis of relative levels (*lower panel*) showed a significant increase in ph-MSK1 and ph-p38 levels and decrease in ph-ERK1/2 levels in tumor compared to resection margin tissues. **b** Representative image of immunohistochemistry (×40) (*left panel*) analysis in paired tissue samples (*n =* 10) and comparison of their relative H-score (*right panel*) showed high ph-MSK1 levels in tumor than resection margin tissues. **c** Immunoblot analysis of AGS cells transiently over-expressing MSK1 showed moderate increase of ph-MSK1 and H3S10ph in corresponding lanes. **d**, **e** Immunoblot analysis of AGS and KATOII cells and immunofluorescence analysis of AGS cells after 6-h treatment with MSK1 inhibitor, H89 (20 μM) showed loss of ph-MSK1 and H3S10ph. **f**, **g** Immunoblot analysis of AGS and KATOII cells and immunofluorescence analysis of AGS cells showed loss of ph-MSK1 and H3S10ph only after 1-h treatment of p38 inhibitor SB203580 (10 μM), but not for ERK1/2 inhibitor PD98059 (10 μM) treatment. *GC* gastric cancer, *RM* resection margin either PRM or DRM with maximum distance from the site of the tumor, *T* tumor, *P* patient. #Fast green-stained PVDF membrane used in Fig. 4d as well. Statistical tests are done by using Wilcoxon matched pairs test. *p < 0.05, **p < 0.01, ***p<0.001

Several kinases are known to phosphorylate H3S10 [12]; however, only mitogen- and stress-activated protein kinase-1 (MSK1)-mediated phosphorylation of H3S10 is known to be involved in cellular transformation [11] which is activated through p38 and/or ERK1/2 MAP kinase pathway [14]. In addition, overexpression of *c-jun* and *c-fos* as observed in our experiments (Fig. 4d) has also been linked to MSK1-mediated phosphorylation of H3S10 at their promoters [15]. Therefore, ph-MSK1, ph-p38, and ph-ERK1/2 levels in tumor and resection margin tissues of GC patients were analyzed. Immunoblot (Fig. 5a, upper panel) as well as its densitometry analysis (Fig. 5a, lower panel) showed the significant increase of ph-MSK1 (*p <* 0.001), p38 (*p <* 0.01), and ph-p38 (*p <* 0.001), while ph-ERK1/2 (*p <* 0.001) levels significantly decrease in tumor compared to resection margin tissues, thus, indicating p38-mediated activation of MSK1 in GC. The increase of ph-MSK1 levels in GC was further confirmed by IHC analysis of the same tissues (Fig. 5b). The observed increase of H3S10ph on the overexpression of MSK1 in AGS cells by immunoblot (Fig. 5c and Additional file 5: Figure S4A) and, moreover, decrease of H3S10ph- on H89-mediated biochemical inhibition of MSK1 by immunoblot studies in AGS and KATOII cell lines (Fig. 5d and Additional file 5: Figure S4B) and immunofluorescence studies in AGS cells (Fig. 5e) confirmed MSK1-mediated phosphorylation of H3S10 in GC. Further, immunoblot analysis with specific antibodies showed a decrease of ph-MSK1 and H3S10ph only on the treatment of p38 inhibitor (SB203580) in AGS and KATOIII cells but not on the treatment of ERK1/2 inhibitor (PD89059) (Fig. 5f). And immunofluorescence studies on inhibitor-treated AGS cells validated that p38 is responsible for phosphorylation of MSK1 in GC (Fig. 5g), thus, confirming p38-MAPK/MSK1-mediated increase of H3S10ph in GC.

## Discussion

In this study on human GC, comparison of several histone PTMs (data not shown) between the tumor and R0 resection margin tissues using immunoblot and IHC resulted H3S10ph with most consistent and significant difference (Fig. 1a, b and Additional file 2: Figure S1). Therefore, H3S10ph was taken for detailed study. To the best of our knowledge, several cell line- and animal model-based studies have shown increase in H3S10ph, as the only histone mark involved in carcinogenesis and cellular transformation [11, 16–18]. However, there is no report on its relative level (tumor vs resection margin) and regulatory pathway in GC. Our IHC analysis in paired samples (*n =* 101), for the first time, demonstrated an increase of H3S10ph in gastric tumor compared to both negative resection margins, PRM and DRM (Fig. 1c–e). This observation also corroborated earlier study in nasopharyngeal carcinoma (NPC) where H3S10ph was found to be significantly higher in the poorly differentiated NPC tissues than normal nasopharynx tissues [17]. On further analysis with clinical parameters, we identified that an increase of H3S10ph in tumor tissues is a marker of poor prognosis and independent prognostic marker for OS in GC (Table 1, Additional file 1: Table S1 and Fig. 2a).

Currently, surgery is the main treatment modality for GC and achieving adequate margin length for R0 resection is a major challenge. With 9–21 % false negative results, palpation, gross inspection, and even assessment of tumor and resection margin by frozen section examination are seemingly unreliable methods to judge the adequacy of resection [19, 20]. Studies in esophageal, pancreatic, rectal, and oral cancer and soft tissue sarcoma have demonstrated that a negative resection margin does not have any prognostic value; however, a positive resection margin and its length affect recurrence and survival of patients [21–25]. The alarmingly high loco-regional recurrence rate in GC patients with R0 resection [26] points towards the fact that a defined negative resection margin is not a “true” negative resection margin. In our study, H3S10ph of both PRM and DRM showed association with clinical parameters and poorly affects OS and DFS (Table 1; Fig. 2b, c). Additionally, H3S10ph levels of DRM showed a positive correlation with recurrence where disease reverted back in 75 % patients in the high-level H3S10ph group compared to 42.5 and 24.6 % in the intermediate and low level H3S10ph groups, respectively. Thus, this study for the first time identified H3S10ph as a potential molecular marker for predicting prognosis of R0 resected GC patients using their histopathologically confirmed negative resection margins. Further, distance-dependent association of H3S10ph with clinical parameters (Table 2) could be utilized in determining the “true” negative resection margin in GC. Demarcation of 4 cm as an optimal margin length in our study rationalizes the recommendations of the National Comprehensive Cancer Network at the molecular level, which state that “the resection margin of more than 4 cm is necessary to achieve a negative microscopic margin” [27]. Therefore, H3S10ph could be helpful in limiting the extent of resection and thereby preventing post-surgery loco-regional recurrence of disease.

The distance-dependent relation of H3S10ph with clinical parameters (Table 2) strongly suggests its association with field cancerization defects. Moreover, various epigenetic factors like chromatin state, histone deacetylase, microRNA, DNA methylation, and chromatin remodeling factors have shown their involvement in field cancerization in a number of cancers including GC [28–31]. Further, earlier in vitro studies have shown that a higher level of H3S10ph alone is directly involved in cellular transformation [11]. Therefore, the occurrence of such epigenetic field defects may facilitate a more permissive chromatin environment for the growth of newly transformed cells. Hence, analysis of high H3S10ph levels in resection margins could predispose the tissue for a high rate GC recurrence after R0 resection.

Most of the earlier reports have shown H3S10ph as a better marker for assessing proliferation and mitotic index than Ki-67 and have also shown increase of H3S10ph as a marker for poor prognosis in several cancers including GC [32–37]. However, except glioblastoma study, none of the cancer studies have used paired normal mucosa or negative resection margin along with tumor tissues; therefore, it is difficult to comment on whether the high proliferation and/or mitotic index or G2/M phase cells is the reason for the increased level of H3S10ph in cancer. H3S10ph is known to regulate protein-protein interactions to favor chromatin condensation as cells enter the M phase, whereas it favors expression of immediate early genes in G1 phase of cell cycle. In light of these cell cycle-specific functions, our data (Fig. 4a–c) have shown no difference in the relative level of cyclins, mitotic index, and cell cycle profile between tumor and paired negative resection margin tissues, thus strongly suggesting that an increase of H3S10ph is independent of G2/M cell cycle phase in GC. A recent report has also shown a cell cycle-independent cigarette side-stream smoke-induced increase of H3S10ph leading to the overexpression of proto-oncogenes, *c-jun* and *c-fos*, and tumor promotion [18]. Further, our study also showed the presence of maximum percentage of cells in the G1 phase of the cell cycle (Fig. 4c), and overexpression of *c-jun* and *c-fos* in tumor compared to paired negative resection margin tissues lead us to believe that the increase of H3S10ph is associated with G1 phase-specific alterations in GC.

Interestingly, global H3S10ph modification levels were lower in “true” negative resection margin tissue and increased significantly in GC. This indicates that the action of the histone-modifying enzymes differs in the resection margin as compared to GC tissues. In our study, a G1 phase-associated increase of H3S10ph and high expression of IE genes, *c-jun* and *c-fos* (Fig. 4), suggest that G1-specific kinase and MSK1 may be phosphorylating H3S10 [12]. Moreover, MSK1 is the only known kinase of H3S10 whose direct role has been implicated in cellular transformation [11, 38]. This notion was further strengthened by the observed high level of ph-MSK1 (an active form of MSK1) in GC tumor tissues (Fig. 5a, b). MSK1 is phosphorylated by MAP kinases, ERK1/2, or p38 in a context-dependent manner [14, 39]. In GC, ph-ERK1/2 has been reported to have no association with clinical parameters [40]. On the other hand, several studies in different cancer like prostate, breast, bladder, liver, lung, transformed follicular lymphoma, and leukemia have suggested a direct role of p38 MAPK in cancer patho-physiological characteristics like proliferation, metastasis, and angiogenesis [41–47]. Further, p38 MAPK being a key regulator of inflammatory response and chronic inflammation is a characteristic of GC which manifests itself by overexpression of pro-inflammatory cytokines like IL-1 and IL-6 [48–50]. Therefore, along with above stated facts, our findings conclude that p38-MAPK/MSK1, but not ERK1/2-MAPK/MSK1, pathway is regulating the H3S10ph in GC (Fig. 5).

## Conclusions

**Fig. 6:**
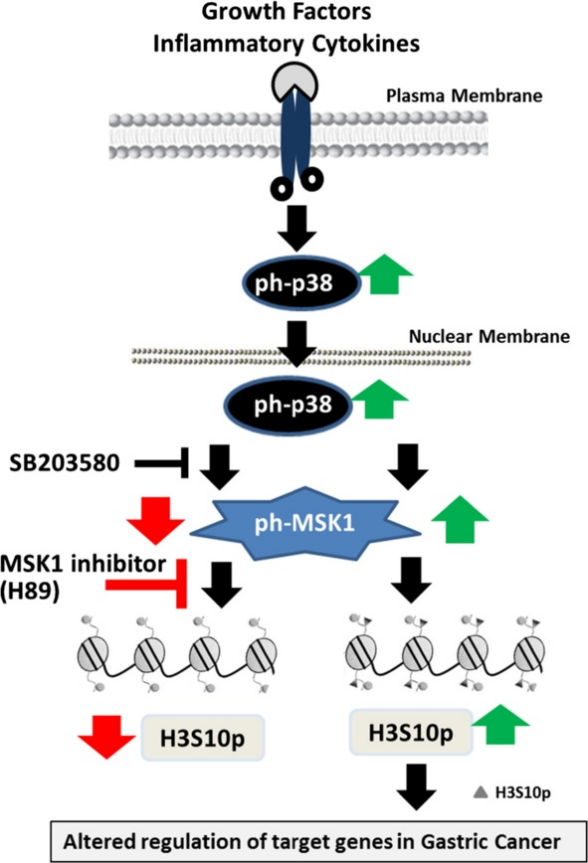
Schematic representation showing p38-MAPK/MSK1 governing upregulation of H3S10ph and MSK1 as a potential target in gastric cancer

In summary, to the best of our knowledge, this study provides the first evidence of a p38-MAPK/MSK1 pathway-regulated increase in H3S10ph with a strong prognostic value in survival as well as in defining the “true” resection margin in GC (Fig. 6). The central role of MSK1-mediated nucleosomal response via H3S10ph in GC might be associated with the induction of aberrant gene expression. Further, the coherence of H3S10ph in GC with two well-known reported altered histone modifications in human cancers, H4K16ac and H3K20me3, suggests that combination of epigenetic modifications may serve as molecular biomarkers for GC. Importantly, our data gave new rationales for using MSK1 as a molecular target along with other epi-drugs to alter the epigenetic landscape in GC for better patient care.

## Additional files

Additional file 1: Figure S1. Densitometry analysis of immunoblot of Figure 1a. Immunoblot was done for indicated antibodies using histone isolated from tumor (T) and negative resection margin (RM) of gastric cancer patients (*n* = 10). Mean intensities of H4K20me3 and H4K16ac were normalized with mean intensity of H4; similarly H3S10ph with H3. Normalized values were represented as relative levels in the form of bar graphs. Statistical tests are done by using Wilcoxon matched pairs test. **p*<0.05, ***p*<0.01, ****p*<0.001. (JPG 64 kb) Additional file 2: Table S1. Survival analysis of variables predicting the risk of death for patients with gastric cancer. (DOCX 30 kb) Additional file 3: Figure S2. Effect of distance of resection margin-dependent H3S10ph levels on GC patients’ survival. Kaplan-Meier survival analysis was done according to H3S10ph staining H-score; low (0–100), intermediate (100–200), and high (200–300). *(A)* and *(B)* Low level of H3S10ph associates with better overall survival (OS) of the patients with PRM ≤4 cm; however it does not affect disease-free survival (DFS). *(C)* and *(D)* Low level of H3S10ph associates with better OS and DFS, however, distance does not affect this association. GC-gastric cancer; PRM-proximal resection margin; DRM-distal resection margin; Int. - Intermediate. Comparison was done by log-rank test. *p* < 0.05 was considered as significant. (TIF 1036 kb) Additional file 4: Figure S3. Effect of 4 cm resection margin distance on GC patients’ survival. Kaplan-Meier survival analysis according to H3S10ph staining H-score: low (0–100), intermediate (100–200), and high (200–300). High level of H3S10P of tumor, PRM, and DRM is associated with both poor overall survival (OS) and disease-free survival (DFS). *(A)* OS and DFS based on H3S10P levels of tumor tissues *(B)* OS and DFS based on H3S10P levels of PRM tissues *(C)* OS and DFS based on H3S10P levels of DRM tissues. Int. - Intermediate. Comparison was done by log-rank test. *p* < 0.05 was considered as significant. (TIF 649 kb) Additional file 5: Figure S4. Cell cycle analysis of AGS cells. Flow cytometry-based cell cycle profile after transfection (A) and H89 treatment (B) showed identical cell cycle profile with negligible apoptosis or cell death. (TIF 311 kb)

## Abbreviations

DFS: Disease-free survival
DRM: Distal surgical resection margin
FFPE: Formalin-fixed paraffin-embedded
GC: Gastric cancer
H3S10ph: Histone H3 serine 10 phosphorylation
IE: Immediate early genes
MSK1: Mitogen- and stress-activated protein kinase-1
NACT: Neoadjuvant chemotherapy
OS: Overall survival
PRM: Proximal surgical resection margin
PTMs: Post-translational modifications
R0: Disease-free surgical resection margin
