# Supplementary figures and images for: p38-MAPK/MSK1-mediated overexpression of histone H3 serine 10 phosphorylation defines distance-dependent prognostic value of negative resection margin in gastric cancer
Source: Clin Epigenetics. 2016 Aug 31;8(1):88. doi: 10.1186/s13148-016-0255-9 (PMC5007744; doi:10.1186/s13148-016-0255-9)

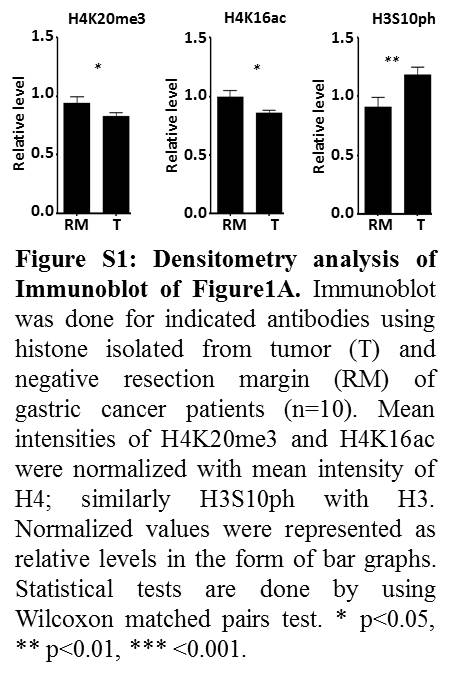

Supplement: Additional file 1: Figure S1. — Densitometry analysis of immunoblot of Figure 1a. Immunoblot was done for indicated antibodies using histone isolated from tumor (T) and negative resection margin (RM) of gastric cancer patients (n = 10). Mean intensities of H4K20me3 and H4K16ac were normalized with mean intensity of H4; similarly H3S10ph with H3. Normalized values were represented as relative levels in the form of bar graphs. Statistical tests are done by using Wilcoxon matched pairs test. *p<0.05, **p<0.01, ***p<0.001. (JPG 64 kb) [file 13148_2016_255_MOESM1_ESM.jpg]

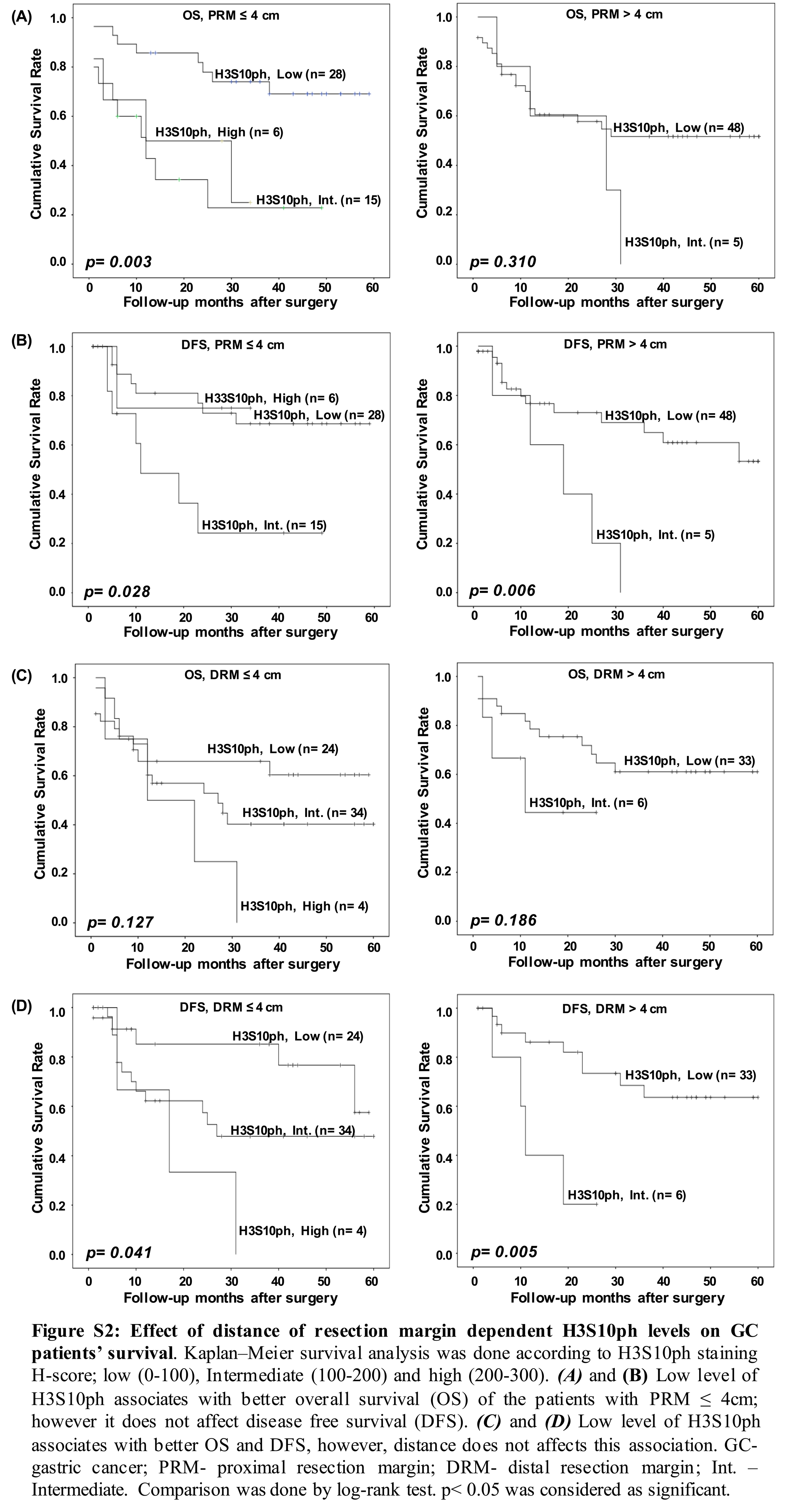

Supplement: Additional file 3: Figure S2. — Effect of distance of resection margin-dependent H3S10ph levels on GC patients’ survival. Kaplan-Meier survival analysis was done according to H3S10ph staining H-score; low (0–100), intermediate (100–200), and high (200–300). (A) and (B) Low level of H3S10ph associates with better overall survival (OS) of the patients with PRM ≤4 cm; however it does not affect disease-free survival (DFS). (C) and (D) Low level of H3S10ph associates with better OS and DFS, however, distance does not affect this association. GC-gastric cancer; PRM-proximal resection margin; DRM-distal resection margin; Int. - Intermediate. Comparison was done by log-rank test. p < 0.05 was considered as significant. (TIF 1036 kb) [file 13148_2016_255_MOESM3_ESM.tif]

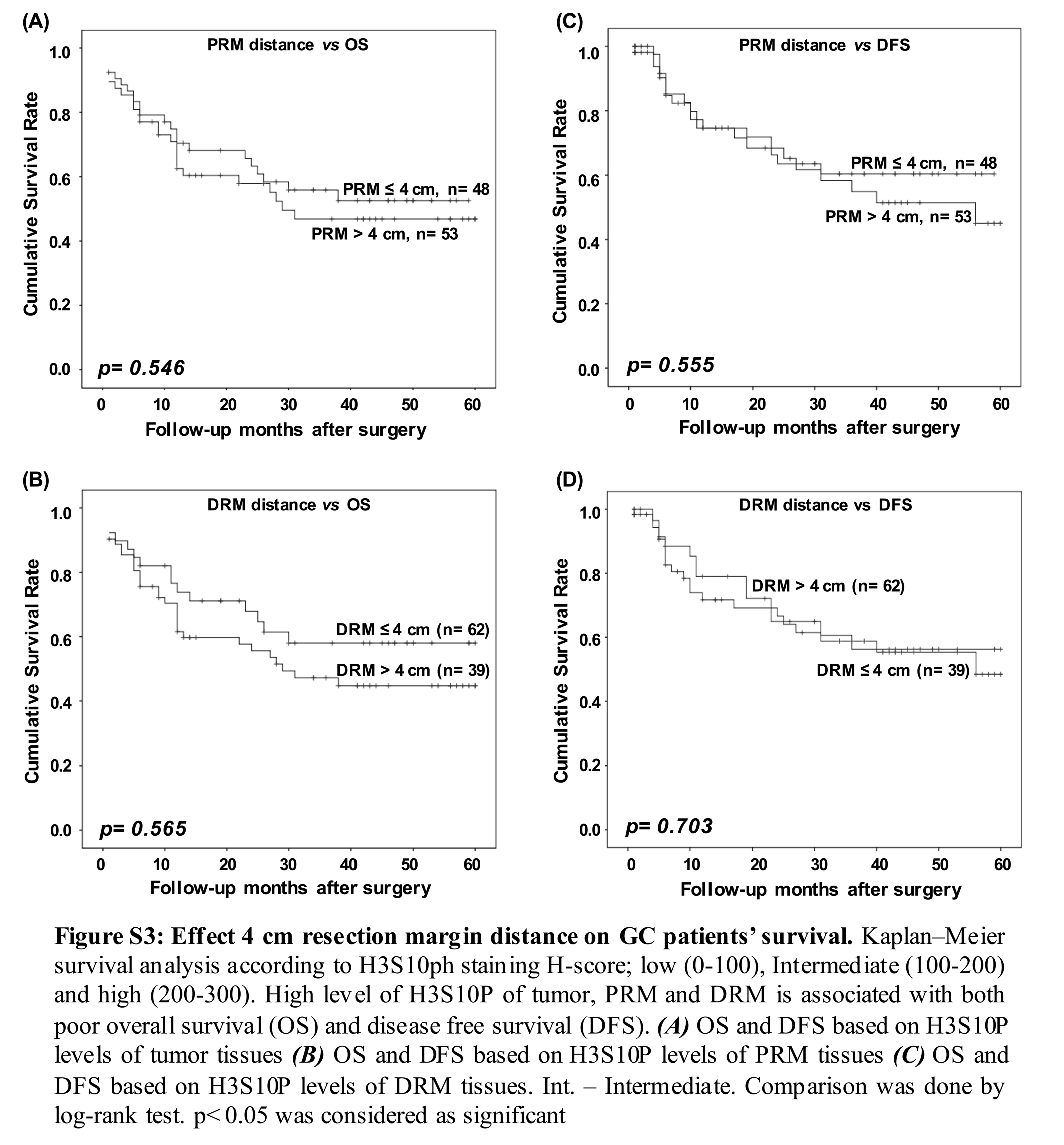

Supplement: Additional file 4: Figure S3. — Effect of 4 cm resection margin distance on GC patients’ survival. Kaplan-Meier survival analysis according to H3S10ph staining H-score: low (0–100), intermediate (100–200), and high (200–300). High level of H3S10P of tumor, PRM, and DRM is associated with both poor overall survival (OS) and disease-free survival (DFS). (A) OS and DFS based on H3S10P levels of tumor tissues (B) OS and DFS based on H3S10P levels of PRM tissues (C) OS and DFS based on H3S10P levels of DRM tissues. Int. - Intermediate. Comparison was done by log-rank test. p < 0.05 was considered as significant. (TIF 649 kb) [file 13148_2016_255_MOESM4_ESM.tif]

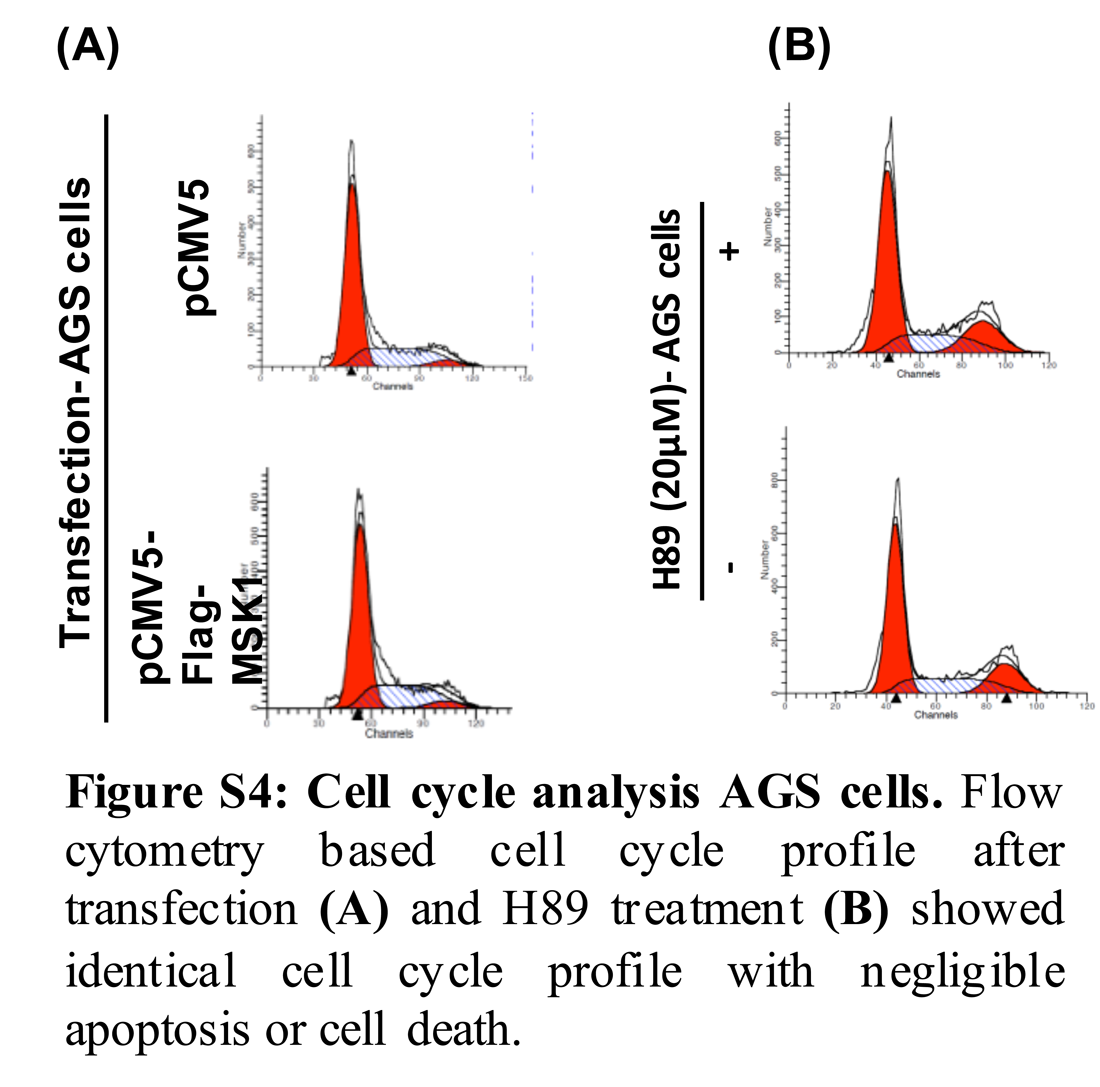

Supplement: Additional file 5: Figure S4. — Cell cycle analysis of AGS cells. Flow cytometry-based cell cycle profile after transfection (A) and H89 treatment (B) showed identical cell cycle profile with negligible apoptosis or cell death. (TIF 311 kb) [file 13148_2016_255_MOESM5_ESM.tif]
