# Supplementary material for: p38-MAPK/MSK1-mediated overexpression of histone H3 serine 10 phosphorylation defines distance-dependent prognostic value of negative resection margin in gastric cancer
Source: Clin Epigenetics. 2016 Aug 31;8(1):88. doi: 10.1186/s13148-016-0255-9 (PMC5007744; doi:10.1186/s13148-016-0255-9)
Supplement: Additional file 2: Table S1. — Survival analysis of variables predicting the risk of death for patients with gastric cancer. (DOCX 30 kb) [file 13148_2016_255_MOESM2_ESM.docx]

| **Table S1: Survival analysis of variables predicting the risk of death for patients with gastric cancer** | | | | | | |
| --- | --- | --- | --- | --- | --- | --- |
| **Variables** | **Overall survival (n= 101)** | | | **Disease free survival (n= 101)** | | |
|  | **Univariate†** | **Multivariate‡** | **HR (CI)** | **Univariate†** | **Multivariate‡** | **HR (CI)** |
| H3S10 phosphorylation status of **Tumor** (Low vs Intermediate vs High) | **0.004** | **0.03** | 2.145  (1.067-4.275) | **0.011** | 0.411 | 1.437  (0.605-3.409) |
| H3S10 phosphorylation status of **PRM** (Low vs Intermediate vs High) | **0.014** | 0.567 | 1.159  (0.700-1.918) | **0.004** | 0.353 | 0.746  (0.402-1.384) |
| H3S10 phosphorylation status of **DRM** (Low vs Intermediate vs High) | **0.026** | 0.592 | 1.2  (0.615-2.344) | **0.006** | 0.402 | 1.393  (0.642-3.025) |
| WHO Classification (WD vs MD vs PD vs SRC) | 0.707 | 0.156 | 0.605  (0.301-1.212 | 0.362 | 0.51 | 1.374  (0.544-3.467) |
| T stage (T1 vs T2 vs T3 vs T4) | 0.062 | 0.375 | 0.783  (0.4561.344) | **0.038** | 0.495 | 1.268  (0.641-2.505) |
| Lymphovascular invasion (Negative vs positive) | **0.011** | 0.115 | 1.719  (0.877-3.3771) | 0.137 | 0.303 | 1.532  (0.681-3.444) |
| Treatment Modality  (Surgery vs NACT+Surgery) | 0.511 | 0.267 | 1.414  (0.767-2.604) | **0.023** | 0.004 | 3.197  (1.460-7.002) |
| pTNM stage  (I vs II vs III vs IV) | 0.062 | 0.169 | 1.614  (0.816-3.191) | **0.038** | 0.654 | 1.214  (0.519-2.842) |
| † Log rank test, ‡ Cox proportional hazard regression, HR- Hazard ration, CI- 95% confidence interval, Bold indicates values that are statistically significant (<0.05). PRM- Proximal resection margin; DRM- Distal resection margin | | | | | | |
